# Role of Race/Ethnicity in Pulmonary Nontuberculous Mycobacterial Disease

**DOI:** 10.3201/eid2103.141369

**Published:** 2015-03

**Authors:** Benjamin S. Thomas, Koh Okamoto

**Affiliations:** Washington University School of Medicine, St. Louis, Missouri, USA (B.S. Thomas);; Rush University Medical Center, Chicago, Illinois, USA (K. Okamoto)

**Keywords:** nontuberculous mycobacterial disease, pulmonary disease, lung disease, epidemiology, race, mycobacteria, bacteria, tuberculosis and other mycobacteria, ethnicity

**To the Editor:** We read with interest the study of gender and age in nontuberculous mycobacterial (NTM) lung disease case-patients in Taiwan (*1*). NTM lung disease is relatively uncommon; however, the exact prevalence of NTM lung disease and causative organisms are largely unknown in many regions of the United States because the disease is not reportable. A recent study using Medicare claims data in the United States showed that the annual prevalence of NTM lung disease increased from 20 cases/100,000 persons in 1997 to 47 cases/100,000 persons in 2007 (*2*). The study also showed that Hawaii had the highest period prevalence of cases (396 cases/100,000 persons), which was at least partially attributed to the large Asian/Pacific Islander population (*2*). During June–December 2011, we conducted a cross-sectional study to evaluate the epidemiologic and clinical significance of NTM isolated from patients in Honolulu, Hawaii; the patients had suspected pulmonary tuberculosis (TB) and were in airborne isolation at a university-affiliated, tertiary-care hospital.

NTM cases were defined according to the 2007 criteria of the American Thoracic Society/Infectious Diseases Society of America (*3*). The process required to establish a diagnosis of NTM lung disease is sometimes lengthy; thus, patients who did not initially meet the disease criteria but who had cultures positive for NTM were reviewed again 1 year after the original data were collected to see if follow-up microbiological and radiographic studies would confirm the presence of NTM lung disease. Descriptive statistics were used to describe categorical and continuous variables. During June–December 2011, a total of 113 patients with suspected pulmonary TB were placed into isolation at the tertiary-care hospital. Of these patients, 85 (75.2%) were men and 28 (24.8%) were women; the median age was 59.8 ± 17 years. Eighteen (15.9%) patients were white, 92 (81.4%) were Asian/Pacific Islander, and 1 (0.9%) was African American; for 2 (1.8%) patients, race/ethnicity was classified as not specified/other.

Of the 113 isolated patients, 21 (18.6%) were positive for mycobacteria. Of these 21 patients, 14 (66.7%) were men and 7 (33.3%) were women; the median age was 64.3 ± 17.3 years. Three (14.3%) of these patients were white, and 18 (85.7%) were Asian/Pacific Islander. *Mycobacterium tuberculosis* and NTM were identified in samples from 3 (14.3%) and 18 (85.7%) of the 21 patients, respectively. Of the 18 patients with NTM-positive samples, 4 (22.2%) had definite NTM lung disease (all of these patients were Asian/Pacific Islander); 2 (11.1%) had probable NTM lung disease; and 12 (66.7%) had possible NTM lung disease. *M. chelonae* (identified by DNA sequencing) was the causative agent for most of the definite cases (n = 3, 75%), and the largest proportion of possible cases was caused by *M. avian* complex bacteria (n = 5, 41.7%).

Our finding that 22.2% (4/18) of the patients in Honolulu with NTM-positive clinical samples during June–December 2011 received a definite diagnosis of NTM lung disease is slightly higher than but consistent with reports from other regions, which show that 9.8%–17.0% of such patients receive a definite NTM disease diagnosis (*4*,*5*). For unclear reasons, the number of NTM disease cases appears to be highest in Asian/Pacific Islander populations. Determining the reason(s) for this discrepancy should be the subject of future research efforts.
